# The Influence of Moisture Content and Workmanship Accuracy on the Thermal Properties of a Single-Layer Wall Made of Autoclaved Aerated Concrete (AAC)

**DOI:** 10.3390/ma18173967

**Published:** 2025-08-25

**Authors:** Maria Wesołowska, Daniel Liczkowski

**Affiliations:** Faculty of Civil and Environmental Engineering, and Architecture, Bydgoszcz University of Science and Technology, S. Kaliskiego 7, 85-796 Bydgoszcz, Poland; danlic002@pbs.edu.pl

**Keywords:** autoclaved aerated concrete (AAC), thermal micro-bridges, thermal insulation, single-layer wall

## Abstract

The use of single-layer aerated concrete walls in residential construction has a tradition of over 60 years. Its main advantage is thermal insulation. It is the most advantageous among construction materials used for the construction of external walls. The possibility of modifying the dimensions of the blocks leads to meeting subsequent restrictive values of the heat transfer coefficient U. The high dimensional accuracy of the blocks allows the use of dry vertical joints and thin joints with a thickness of 1–3 mm, the thermal influence of which is omitted. However, the thermal uniformity of such a wall is strictly dependent on the quality of workmanship. The main objective of the analysis is to assess the impact of moisture on the *U*_wall_ of walls as a function of vertical joint spacing and horizontal joint thickness. It should be said that the effect of humidity and manufacturing accuracy on the thermal properties of aerated concrete walls has not been sufficiently studied. Further study of these patterns is necessary. Particular attention should be paid to the thin-bed mortar, which depends on the manufacturing accuracy. The separation of AAC masonry elements that occurs during bricklaying significantly affects the thermal insulation of walls. This issue has not yet been analysed. The scientific objective of this article is to develop a procedure for determining the thermal properties of a small, irregular air space created as a result of the separation of masonry elements and the impact of this separation on the thermal insulation of the wall. Based on the analysis of the thermal conductivity of voids and masonry elements, it was determined that this impact is visible at low AAC densities. A detailed analysis taking into account both these joints and horizontal joints, as well as different moisture levels, made it possible to determine the permissible separation of AAC blocks, at which the high thermal insulation requirements applicable in most European countries are met. The analysis showed that it is possible to meet the thermal protection requirements for 42 cm wide blocks intended for single-layer walls with a maximum vertical contact width of 3 mm and a joint thickness of up to 2 mm. AAC moisture content plays a major role in thermal insulation. Insulation requirements can be met for AAC in an air-dry state, as specified by ISO 10456.

## 1. Introduction

The use of single-layer aerated concrete walls in residential construction has a tradition dating back over 60 years. Worldwide, approximately 30% of buildings are constructed from autoclaved aerated concrete [1]. AAC is mainly used for erecting walls, ceilings and flat roofs, with masonry walls made of small-size elements accounting for the largest share [2]. These elements are mainly produced in density classes 600, 500 and 400. For single-layer walls, systems based on masonry elements made of the lightest types of aerated concrete (300, 350 and 400 kg/m^3^) [3] are used. Research has shown that aerated concrete in this density range is characterised by an almost linear relationship between thermal insulation and density [4], which makes it possible to construct single-layer walls that meet thermal requirements while complying with structural safety, fire safety and noise protection conditions [5]. The requirements for the performance characteristics of AAC are contained in standard EN 771-4 [6]. The standard does not impose any requirements on shape, dimensions, density, strength or thermal conductivity. However, it specifies a maximum dry density of 1000 kg/m^3^ and a minimum compressive strength for use in load-bearing elements (1.5 N/mm^2^). The λ-values of AAC usually range from 0.07 to 0.2 W/(mK) in a ρ interval between 300 and 750 kg/m^3^ [7,8,9,10,11].

Thermal properties are specified in EN 1745 [12]. Manufacturers declare λ_10,dry,mat_ using this standard or by performing thermal conductivity tests (Table 1).

The performance parameters of modern masonry elements have been improved by the following [13,14]:

- Modification of the composition to achieve more favourable thermal properties;
- New ergonomic shapes of elements (vertical locks and grip holes);
- Use of new joining techniques for thin joints or warm mortars, enabling the construction of energy-efficient buildings [15].

The *U*_wall_-value is a key parameter describing the thermal insulation properties of insulating and structural walls [16]. The required maximum U-value depends on national regulations. In European countries, it is approximately 0.20 W/m^2^K. This requires the use of low-density blocks with a width of ≥42 cm.

An important issue in the construction of single-layer energy-efficient walls is not only the quality of the products themselves but also the workmanship of the walls. The required accuracy for masonry elements and the methods for determining them are specified in standards [12,17,18]. Information on masonry methods is provided by manufacturers in their implementation guidelines [19].

When using blocks with tongue-and-groove profiled-end faces (*p* + W), masonry is laid using the so-called dry joint method, whereby blocks cut and laid at wall joints (e.g., in building corners or where walls are connected to each other) should be joined by filling the vertical joint with masonry mortar [20]. Vertical joints of profiled masonry elements up to 3 mm wide are considered acceptable, with a negligible effect on the thermal properties of the wall. Above 3 mm, they should be filled with heat-insulating mortar [20].

Both butt joints and gaps in vertical locks constitute material thermal bridges, which, due to their dimensions (in the order of mm), can be called thermal micro-bridges [21].

Numerical modelling of heat transfer for AAC walls focuses on three basic aspects:

- Heat transfer through porous material [21];
- Heat transfer through mortar-filled joints (mainly horizontal) [22,23,24];
- Heat transfer through typical thermal bridges in external walls [25,26].

There is a lack of studies on unfilled vertical joints.

The aim of the analysis is to

- Develop a procedure for determining the thermal properties of a small, irregular air space created by the separation of masonry elements and the impact of this separation on the thermal insulation of the wall;
- Determine the impact of the thickness of a thin horizontal joint on the thermal insulation of the wall;
- Introduce AAC thermal moisture into the analysis, which was taken into account by assuming five calculation variants, differing in moisture content in the material from 0 kg/kg to 0.08 kg/kg.

The impact of moisture on the heat transfer coefficient U of the wall was assessed as a function of joint spacing and joint thickness. The key reference point is the permissible joint spacing of 3 mm, but the analysis also includes a comparison of U values for joint spacings exceeding 3 mm (hypothetical gaps of 5 and 7 mm). The analysis took into account different weld thickness variants not exceeding 3 mm (value resulting from dimensional tolerance).

## 2. Design Values of the Thermal Conductivity Coefficient

The thermal and moisture properties of autoclaved aerated concrete (AAC) specified in manufacturers’ declarations of performance (DOP) mainly include thermal conductivity in dry conditions [27] and general data on specific heat and the water vapour diffusion resistance coefficient (tabular values based on EN 1745 [12] and ISO 10456 [28]). The current standardisation introduces two concepts relating to the thermal conductivity coefficient of materials:

- Declared value (*λ_D_*), used for production quality control, corresponding to laboratory conditions;
- Design value (*λ*), used for design purposes, corresponding to the conditions of use of the material in the building.

**Figure 1:**
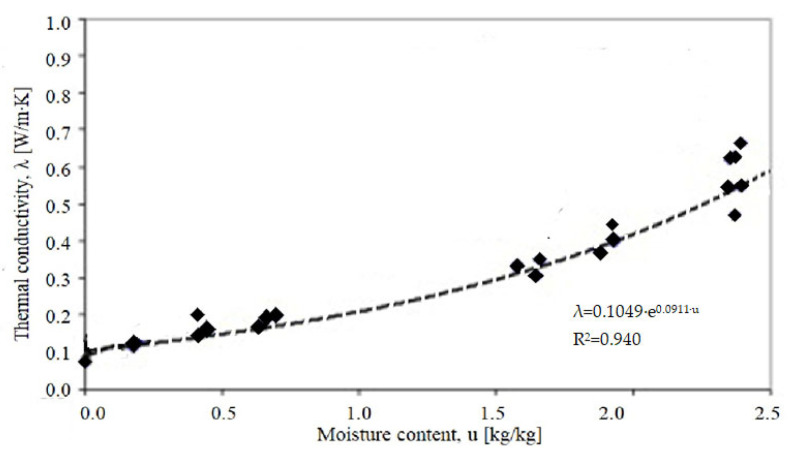
Dependence of the AAC300 thermal conductivity coefficient (λ) on capillary moisture content (u) (own study based on [31]).

The increase in moisture content in the material resulting from its operation in appropriate environmental conditions has a significant impact on the thermal conductivity coefficient. Zapotoczna-Sytek indicates that the maximum moisture content by weight due to frost damage should not exceed 30% of the mass [15]. The stabilised moisture content is 5–8% by weight [28,29,30], which is achieved in properly operated buildings after 2–3 years [28]. The results of experiments by Jerman et al. indicate that the design value of the thermal conductivity coefficient in construction applications can be up to six times higher in a state of capillary water saturation than in dry conditions and up to 50% higher when the temperature rises from 2 °C to 40 °C [31]. The effect of AAC moisture content on its thermal properties has been the subject of numerous laboratory studies [29,30,31,32]. Sample tests for a density of 300 are shown in Figure 1. Ultimately, however, the results are compared with standard values and converted according to the procedures of ISO 10456 [28].

Determining the design value involves taking into account the differences in humidity between the conditions for which the declared thermal conductivity value was determined and the conditions in which the material actually operates. At the design stage, the operating conditions of the material should be predicted and the declared thermal conductivity coefficient *λ*_*D*_ converted to the design value *λ*:

(1) $$\lambda =\lambda_{D}\cdot F_{T}{\cdot F}_{m}$$

where *λ* is the calculated value of the thermal conductivity coefficient AAC, *λ_D_* is the declared value of the AAC thermal conductivity coefficient, and *F_T_* is the temperature conversion factor:

(2) $$F_{T}=e^{f_{T}\cdot \left(T_{2}-T_{1}\right)}$$

In the case of building insulation, in most cases, the *F_T_* temperature conversion factor is of secondary importance, and, with some approximation, its value can be assumed to be 1. However, the heat transfer coefficient values are declared at 10 °C (λ_dry,10_ [12]), and for single-layer walls, the temperature in the wall varies between −18 and +20 °C. In this case, the proposed conversion level of 1 is too much of a simplification.

*F_m_* is the moisture conversion factor taking into account the mass moisture content characteristic of the AAC material:

(3) $$F_{m}=e^{f_{u}\cdot \left(u_{2}-u_{1}\right)}$$

where

$f_{u}$ is the conversion factor due to moisture as the ratio of mass to mass $\left[\frac{kg}{kg}\right]$;

$u_{1}$ is the moisture content as a mass ratio for the first set of conditions $\left[\frac{kg}{kg}\right]$ (dry state for determining the declared value, *u*_1_ = 0.0 kg/kg);

$u_{2}$ is the moisture content as a mass ratio for the second set of conditions $\left[\frac{kg}{kg}\right]$ (for design conditions). 

According to ISO 10456, autoclaved aerated concrete with a density range of 300–1000 kg/m^3^ is characterised by a hygroscopic moisture content

At a temperature of +23 °C and 50%RH: $u_{2}$= 0.026 kg/kg;

At a temperature of +23 °C and 80%RH: $u_{2}$= 0.045 kg/kg [28].

The conversion factor due to moisture content in the range of 0–0.25 kg/m^3^ is *f_u_* = 4.

## 3. Materials and Methods

The subject of the study is a 42 cm thick insulating and structural wall made of AAC 300.

**Figure 2:**
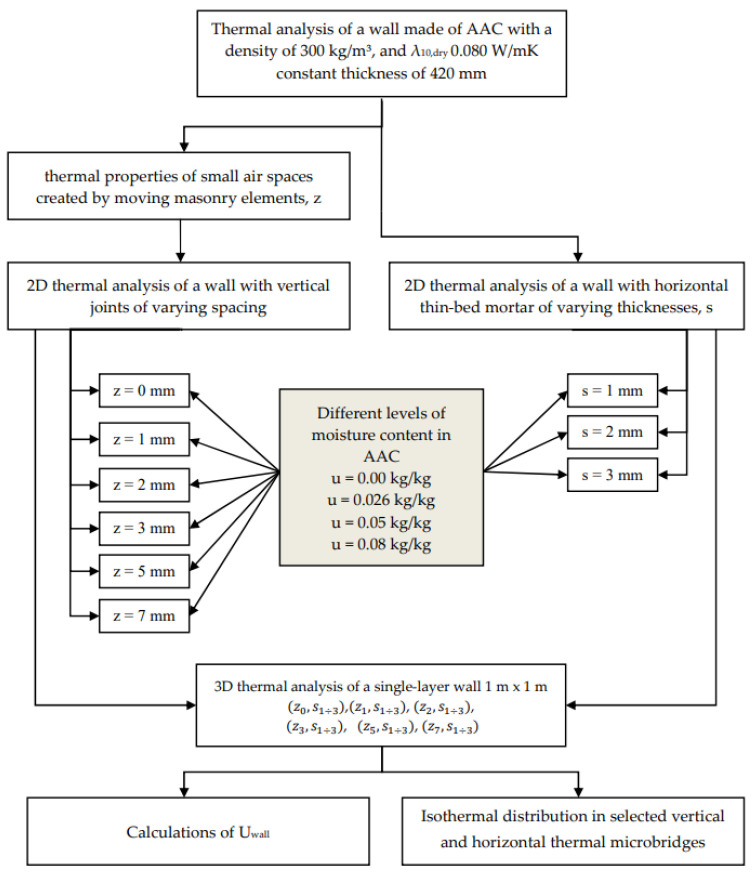
Block diagram of experimental research.

The thermal conductivity was assumed to be λ_10,dry,mat_ = 0.08 W/mK (Table 1). The diagram of experimental work is presented in Figure 2.

The calculation procedures for the heat transfer coefficient (U) and the corrected heat transfer coefficient ($U_{C}$) contained in the EN ISO 6946 [33] standard do not describe the impact of air voids created by the construction of insulating and structural walls with vertical locks. Such walls should be analysed using the numerical procedure according to EN 1745 [12], which allows for the consideration of both horizontal and vertical joints and voids generated by grip holes.

**Figure 3:**
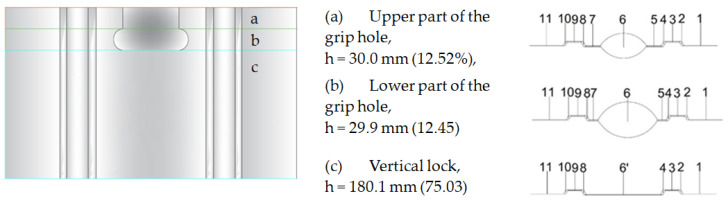
Shape of the gap in characteristic cross-sections of vertical contact between elements with AAC.

Due to the dimensions and profiling of the blocks, the joint forms a gap with a complex shape (Figure 3). Depending on the spacing of the blocks, the areas 1–11 marked in Figure 3 change their geometry. Consequently, the thermal properties also change.

**Figure 4:**
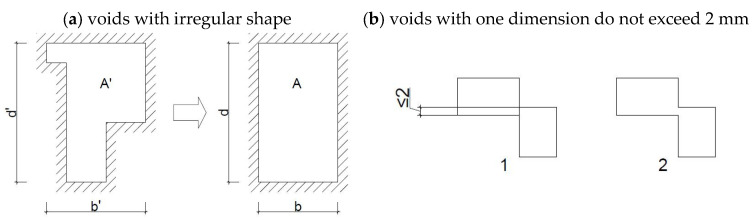
The ISO 10077-2 standard’s approach to closed air voids [34]. A—equivalent surface area of a rectangular air void; d, b—depth and width of equivalent air void; A′—area of the actual void; d′, b′—the depth and width of the smallest rectangle inscribed.

Due to the irregular shape of the voids, the procedures of the EN ISO 10077-2 [34] standard were applied, according to which voids with one dimension do not exceed 2 mm or voids whose mutual connection does not exceed 2 mm are considered separate, as shown in Figure 4b. Non-rectangular air ducts are converted into rectangular ones with the same area (A = A′) and shape factor (d/b = d′/b′)—Figure 4a.

**Figure 5:**
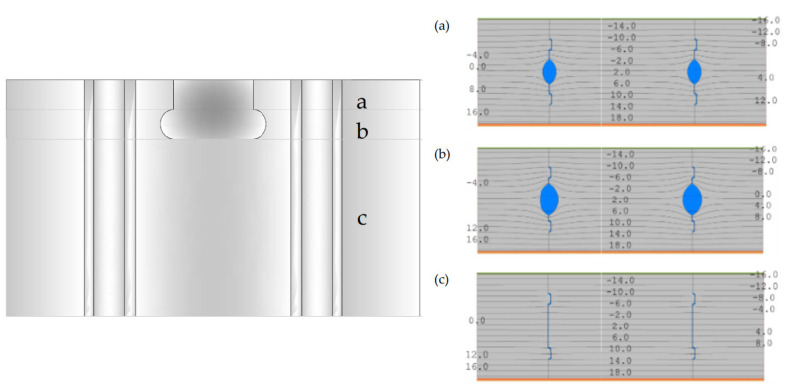
Temperature distribution in a single-layer wall with AAC in characteristic vertical contact sections: (**a**) upper part of the grip hole, h= 30.0 mm (12.52%); (**b**) lower part of the grip hole, h = 29.9 mm, 12.45%; (**c**) vertical lock, h = 180.1 mm, 75.03%.

In order to determine the equivalent thermal conductivity of cavities, it is necessary to know the temperature distribution within the wall thickness. For this purpose, a reference model without a gap in the vertical joint was adopted. The values of $T_{m}$ and Δ*T* were determined for three characteristic cross-sections of the model (Figure 5) based on calculations in the Therm programme [35] using the boundary conditions t_i_ = +20 °C, t_e_ = −18 °C. 

**Figure 6:**
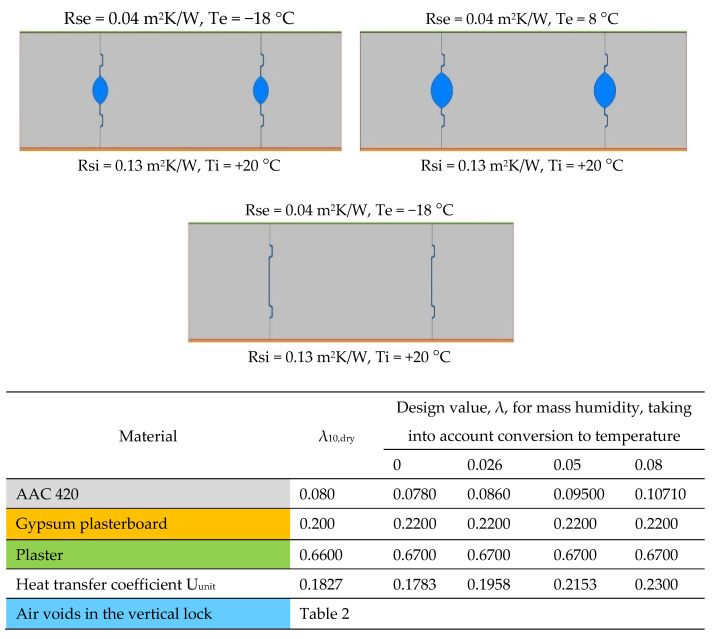
Calculation models for vertical contact (own study based on [18,27,36,37]).

**Figure 7:**
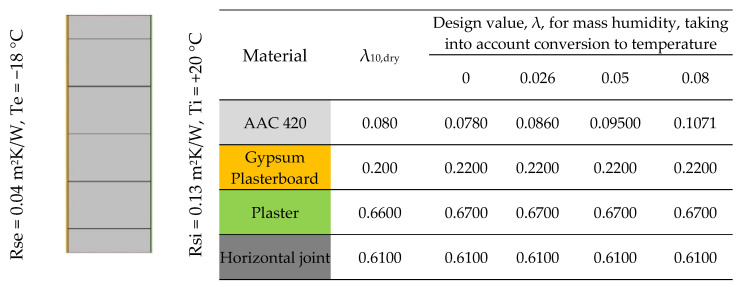
Calculation model for horizontal joints (own study based on [27,36,37,38]).

Six 2D models were created with air gaps in the vertical joint of widths 0 mm, 1 mm, 2 mm, 3 mm, 5 mm and 7 mm. The values of ∆T and $T_{m}$ were determined, then λ_eq_ (Table 2). The calculation models of vertical joints are shown in Figure 6. For horizontal joints, three 2D models were created with mortar thicknesses of 1 mm, 2 mm and 3 mm (Figure 7). Using these models, linear heat transfer coefficients for a single joint and heat transfer coefficients for these cases were determined using Therm [35]. Based on the results obtained, heat transfer coefficients were determined for each model. As a reference point, heat transfer coefficients were determined for structures without joints, taking into account only the conversion from temperature and moisture for the materials forming the wall.

Six 2D models were created with air gaps in the vertical joint of widths 0 mm, 1 mm, 2 mm, 3 mm, 5 mm and 7 mm. The values of ∆T and $T_{m}$ were determined, then λ_eq_ (Table 2). The calculation models of vertical joints are shown in Figure 6. For horizontal joints, three 2D models were created with mortar thicknesses of 1 mm, 2 mm and 3 mm (Figure 7). 

Using these models, linear heat transfer coefficients for a single joint and heat transfer coefficients for these cases were determined using Therm [35]. Based on the results obtained, heat transfer coefficients were determined for each model. As a reference point, heat transfer coefficients were determined for structures without joints, taking into account only the conversion from temperature and moisture for the materials forming the wall.

**Figure 8:**
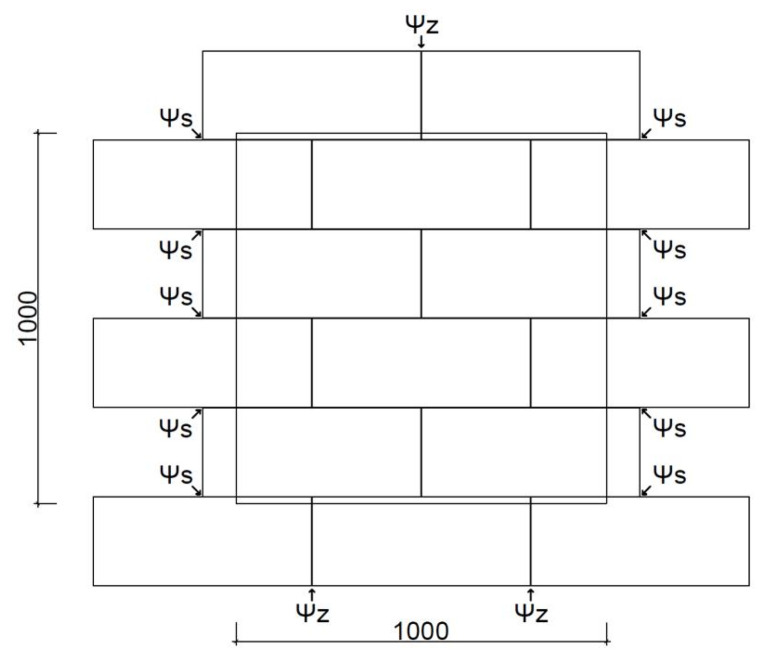
Calculation model for determining the heat transfer coefficient of a single-layer wall.

After obtaining the linear heat transfer coefficients for joints (*z*_1÷7_) and welds (*s*_1÷3_), variations of these contacts were made on a 1 m × 1 m section of the wall, resulting in eighteen models, $\left(z_{0},s_{1÷3}\right)$, $\left(z_{1},s_{1÷3}\right)$, $\;(z_{2},s_{1÷3})$, $\;(z_{3},s_{1÷3})$, $\;(z_{5},s_{1÷3})$, and $\;(z_{7},s_{1÷3})$, for each of the five variants of the thermal conductivity coefficient for the masonry element. For each thermal bridge, the values of the linear heat transfer coefficient, the heat transfer coefficient and the final heat transfer coefficient for 1 m^2^ of wall were determined (Figure 8). The direct heat transfer coefficient through transmission between the heated space and the external environment through the building envelope [39] was determined as follows

(4) $$H_{d}=A\cdot U_{0}+\Psi_{z}\cdot \sum_{z}l_{z}+\Psi_{s}\cdot \sum_{s}l_{s}$$

where

A is the surface area of the element $\left[m^{2}\right]$;

$U_{0}$ is the heat transfer coefficient for a cross-section through a masonry element $\left[\frac{W}{m^{2}\cdot K}\right]$;

$\Psi_{z}$ is the linear heat transfer coefficient of the thermal bridge of joints $\left[\frac{W}{m\cdot K}\right]$;

$l_{z}$ is the length of the linear thermal bridge of the joint $\left[m\right]$;

$\Psi_{s}$ is the linear heat transfer coefficient of the thermal bridge of the weld $\left[\frac{W}{m\cdot K}\right]$;

$l_{s}$ is the length of the linear thermal bridge of the weld $\left[m\right]$.

The resulting coefficient for the wall is

(5) $$U_{wall}=\frac{H_{d}}{A}\left[\frac{W}{m^{2}\cdot K}\right]$$

## 4. Research Results and Discussion

### 4.1. Heat Transfer Coefficient

**Figure 9:**
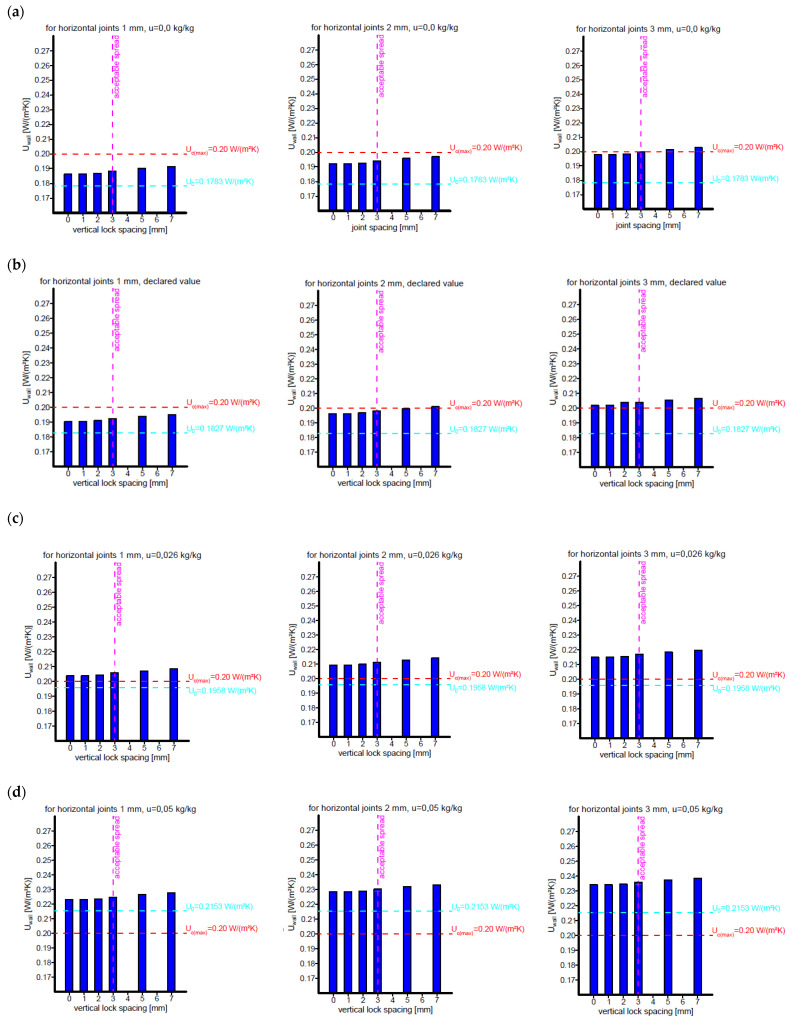

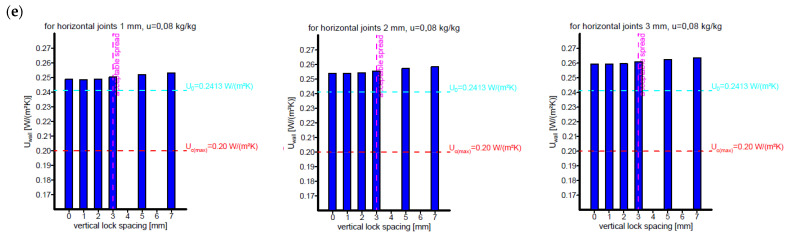
Changes in the heat transfer coefficient for the AAC wall as a function of the vertical contact spacing and joint thickness. (**a**) Calculation variant 1: Temperature conversion of wall materials, AAC moisture content u = 0.00 kg/kg. (**b**) Calculation variant 2: Declared values of thermal conductivity coefficients. (**c**) Calculation variant 3: Temperature and humidity conversion of wall materials, AAC humidity u = 0.026 kg/kg (standard value for RH = 50% [40]). (**d**) Calculation variant 4: Temperature and humidity conversion of wall materials, AAC humidity u = 0.05 kg/kg (stabilised value according to [28]). (**e**) Calculation variant 5: Temperature and humidity conversion of wall materials, AAC humidity u = 0.08 kg/kg (stabilised value according to [40]).

**Figure 10:**
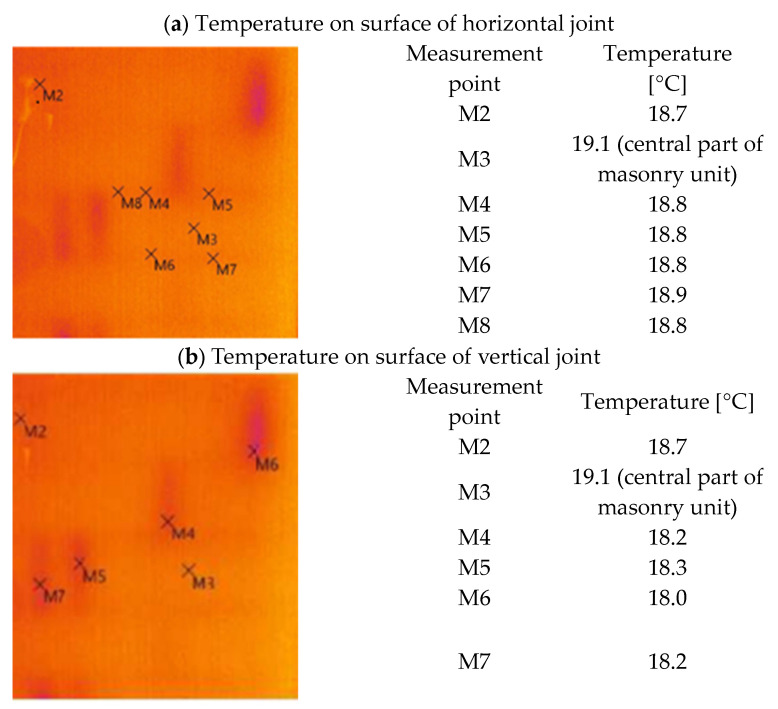
Thermogram of a single-layer wall with AAC (own study).

The results of the tests are summarised in Figure 9 and Figure 10, where *U*_0_ is the heat transfer coefficient for a cross-section through a masonry element and U_c(max)_ is the permissible value.

### 4.2. Temperature on Surface

The surface temperature was used to compare the adopted calculation model for vertical joints and connections. Thermal images of the actual wall (Figure 10) and isotherms in the calculation models of masonry with a connection and masonry with a horizontal joint were taken.

Thermal imaging tests performed on the actual object indicate that the quality of the vertical joint has a significant impact on heat transfer:

- The temperature difference on the inner surface of the wall between the surface of the block and the surface above the horizontal joint was Δt = 18.1−17.8 = 0.3 °C.
- The temperature difference on the inner surface of the wall between the block surface and the plaster surface above the vertical joint was Δt = 18.1 −(17.0 ÷ 17.3) = (1.1 ÷ 0.8) °C.

**Figure 11:**
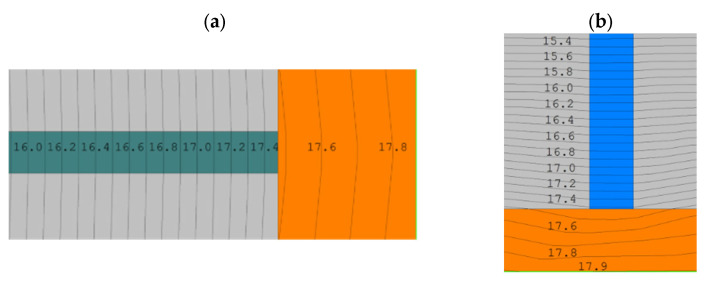
Temperature distribution in thermal micro-bridges: (**a**) 7 mm vertical joint; (**b**) 3 mm horizontal thin-bed mortar.

The downward trend in temperatures is also evident in the heat transfer calculations for the vertical and horizontal joints (Figure 11).

## 5. Discussion

For all five variants, the *U*_wall_ increases with increasing joint spacing and weld thickness, with the increase being most noticeable for larger joint spacing (5 mm and 7 mm). Joint spacing is one of the important factors affecting the *U*_wall_, as it increases the proportion of thermal bridges in the partition. Linear heat transfer coefficients are in the order of a few thousandths of W/mK, but their total length in 1 m^2^ of a wall surface reaches up to 1.5 m, which, combined with welds with a total length of 5 m, gives values at the level of

Variant 1, 2, 3: ΔU ≈ 0.01–0.020 W/m^2^K;

Variant 4: ΔU ≈ 0.01–0.025 W/m^2^K;

Variant 5: ΔU ≈ 0.01–0.030 W/m^2^K.

An analysis of the above trends indicates that the effect of joint spacing and weld thickness on the increase in *U*_wall_ is non-linear and intensifies with increasing AAC moisture content. In particular, for variant 5, a significant increase in the heat transfer coefficient is noticeable, suggesting that further increases in these parameters lead to unacceptable heat losses. The increase in the *U*_wall_ is monotonic with respect to the joint spacing and joint thickness in all five cases, suggesting a strong correlation between these parameters and the reduction in the thermal insulation properties of the partition.

The assumed thickness of a single-layer wall meets the thermal protection requirements only for variants 1 and 2 in terms of the permissible vertical contact width (3 mm) and joint thickness up to 2 mm (Figure 9a,b). Exceeding these dimensions results in exceeding the assumed value of U_c(max)_ = 0.20 W/m^2^K.

Taking into account the standard conversion for humidity allows the thermal protection requirements for the cross-section through the block to be met, while taking into account joint and connection results in the wall *U*_wall_ being exceeded by 0.05–0.15 W/m^2^K for permissible joint thicknesses and contact widths (Figure 9c).

The stabilised moisture content ranges of 5–8% indicated in the literature cause the heat transfer coefficient to be exceeded for the cross-section through the block and further exceedances resulting from contacts and joints (for z ≤ 3 mm and s ≤ 3 mm), as shown in Figure 9d,e:

- For humidity 5%, *U*_0_ = 0.2153 W/m^2^K, *U*_wall_ = 0.223–0.239 W/m^2^K;
- For humidity 8%, *U*_0_ = 0.230 W/m^2^K, *U*_wall_ = 0.249–0.264 W/m^2^K.

The isothermal distribution obtained in the developed models corresponds to the temperature differences recorded on the thermograms. The spaced vertical joints and horizontal thin-bed mortar are thermal micro-bridges that significantly affect the temperature distribution and the heat transfer coefficient. However, from the obtained temperature differences, it appears that the guidelines for wall masonry were not strictly followed: it can be assumed that there are horizontal joints with a greater thickness than that recommended by the manufacturer and vertical joints that do not ensure contact between the blocks.

Despite the detailed approach to modelling small air spaces and material thermal micro-bridges, it should be noted that the adopted geometric models and physical parameter values are subject to a certain degree of simplification and uncertainty. This applies in particular to the geometry of joints, the accuracy of material layer dimensions and the assumed values of the thermal conductivity coefficient and its conversion due to humidity. As presented in the article, much of the data often comes from the literature or catalogues and may not reflect actual in situ conditions.

Therefore, it would be useful to conduct a sensitivity analysis to assess the extent to which the variability of selected parameters affects the final result of the U-value calculation. Such an analysis would allow the identification of the parameters with the greatest impact on the final result and a better estimation of the uncertainty associated with the numerical model. In future stages of the research, it is planned to address this issue by applying probabilistic methods or Monte Carlo simulations [41].

## 6. Conclusions

The results indicate that when designing structural connections in building partitions, not only the *U*_0_ value should be taken into account but also the influence of the joint geometry and weld thickness on the final thermal parameters of the wall. Due to the actual stabilised humidity emphasised in many studies, the thermal conductivity coefficient should be converted. An increase in the thermal conductivity coefficient results in a single-layer wall thickness deficit of up to 10 cm (at a stabilised humidity of 8%).

For gaps up to 3 mm, the increase in the U-value is moderate, above 3 mm, which is significant. This is due to the fact that with larger gaps, local areas of increased thermal conductivity appear, which cause a non-linear increase in the *U*_wall_. This effect is particularly significant at higher humidity values, which means that in operating conditions with high humidity, the gap between the joints should be minimised.

The use of smaller joint thicknesses (1 mm) allows for better control of the *U*_wall_ and minimises the negative impact of joint spacing. For thicker joints (2 mm and 3 mm), the increase in the *U*_wall_ is more pronounced, and exceeding the limit value at greater spacing may lead to the need for additional thermal insulation. The thickness of the joint is important in conditions of increased humidity—the thicker the joint, the greater the increase in *U*_wall_, especially for gaps greater than 3 mm.

The results of the analysis indicate that the humidity of building materials and the geometry of masonry joints have a significant impact on the heat transfer coefficient. The effect of the increase in *U*_wall_ is particularly pronounced in the case of a combination of large joint gaps and high humidity, which suggests the need to minimise these parameters in the design of energy-efficient building partitions.

The analysis showed that for an AAC density of 300 kg/m^3^, the permissible spacing between blocks is 3 mm and the maximum joint thickness is 3 mm. An important factor is the moisture content of the AAC. The blocks must be kept in a dry air condition, which is only possible with high-quality workmanship (protection against moisture during storage and masonry) and protection of the surface of the finished wall with plaster that protects against environmental moisture.

Obtaining a dry vertical joint requires the appropriate masonry technique by sliding the next block into the lock from above rather than pushing the block in, as is the case in traditional masonry when vertical joints are filled.

## Figures and Tables

**Table 1:** Thermal properties of autoclaved aerated concrete.

Density of the Material (Net Dry Density)	λ_10,dry,mat_ According to EN 1745 [12] [W/(m·K)]		λ_10,dry,mat_ According to Declaration of Performance of Selected Manufacturers [W/(m·K)]			
[kg/m^3^]	*p* = 50%	*p* = 90%	Manuf. 1	Manuf. 2	Manuf. 3	Manuf. 4
300	0.072	0.085	0.075 (according to EN 1745)	0.072 (S1, *p* = 50%)	0.085 (S2, *p* = 90%)	0.080 (S2, *p* = 90%)
400	0.096	0.110	AAC densities for which it is not possible to meet thermal protection requirements with a wall thickness of 42 cm			
500	0.120	0.130				
600	0.150	0.160				
700	0.170	0.180				
800	0.190	0.210				
900	0.220	0.240				
1000	0.240	0.260				

**Table 2:** Values of λ_eq_ for the assumed variants of the gap in the vertical contact of the AAC wall according to the calculation procedures [33,34].

Joint Geometry			Voids in the Joint										
			1	2	3	4	5	6	7	8	9	10	11
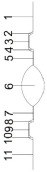	∆T[K]		6.9	0.9	3.0	1.0	4.2	4.2	4.2	1.0	2.9	1.0	6.9
	$T_{m}[K]$		15.15	11.25	9.3	7.3	4.7	0.5	−3.7	−6.3	−8.25	−10.15	−14.15
	λ_eq_ for joint separation, W/mK	0 mm	-	0.058	0.092	0.061	0.097	0.419	0.097	0.061	0.092	0.058	-
		1 mm	0.203	0.061	0.097	0.065	0.098	0.369	0.092	0.059	0.085	0.054	0.154
		2 mm	0.204	0.064	0.098	0.067	0.099	0.370	0.093	0.061	0.085	0.056	0.155
		3 mm	0.206	0.067	0.107	0.068	0.114	0.371	0.108	0.062	0.093	0.058	0.156
		5 mm	0.287	0.071	0.109	0.073	0.116	0.373	0.110	0.067	0.095	0.062	0.237
		7 mm	0.290	0.076	0.111	0.077	0.118	0.374	0.111	0.070	0.097	0.065	0.239
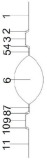	∆T[K]		7.3	1.1	3.2	1.3	3.1	4.2	3.1	1.3	3.2	1.1	7.3
	$T_{m}[K]$		14.95	10.75	8.6	6.35	4.15	0.5	−3.15	−5.35	−7.6	−9.75	−13.95
	λ_eq_ for joint separation, W/mK	0 mm	-	0.058	0.092	0.061	0.069	0.535	0.069	0.061	0.092	0.058	-
		1 mm	0.203	0.061	0.097	0.064	0.070	0.343	0.066	0.059	0.085	0.054	0.155
		2 mm	0.204	0.064	0.098	0.067	0.071	0.472	0.067	0.062	0.086	0.056	0.155
		3 mm	0.205	0.066	0.107	0.067	0.072	0.473	0.068	0.062	0.095	0.058	0.156
		5 mm	0.289	0.071	0.109	0.073	0.074	0.475	0.070	0.067	0.097	0.062	0.239
		7 mm	0.291	0.075	0.111	0.077	0.075	0.476	0.072	0.070	0.099	0.065	0.241
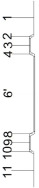	∆T[K]		6.6	0.9	2.7	1.1	-	13.8	-	1.1	2.7	0.9	6.5
	$T_{m}[K]$		15.4	11.65	9.85	7.95	-	0.5	-	−6.95	−8.85	−10.65	−14.35
	λ_eq_ for joint separation, W/mK	0 mm	-	0.058	0.092	0.061	-	0.386	-	0.061	0.092	0.058	-
		1 mm	0.204	0.061	0.098	0.065	-	0.371	-	0.059	0.084	0.054	0.154
		2 mm	0.205	0.064	0.099	0.067	-	0.372	-	0.061	0.085	0.056	0.155
		3 mm	0.206	0.067	0.106	0.068	-	0.646	-	0.061	0.092	0.058	0.156
		5 mm	0.286	0.072	0.108	0.074	-	0.648	-	0.066	0.094	0.061	0.235
		7 mm	0.289	0.076	0.111	0.078	-	0.650	-	0.070	0.096	0.064	0.236
Δ*T* and *T_m_* according to Figure 5.													

## Data Availability

The original contributions presented in this study are included in the article. Further inquiries can be directed to the corresponding author.
